# 2-Ureido-1,3-thia­zol-3-ium dihydrogen phosphate

**DOI:** 10.1107/S1600536811021337

**Published:** 2011-06-11

**Authors:** Kateryna Gubina, Iuliia Shatrava, Vladimir Ovchynnikov, Vladimir Amirkhanov

**Affiliations:** a National Taras Shevchenko University, Department of Chemistry, Volodymyrska Street 64, 01033 Kyiv, Ukraine

## Abstract

The title compound, C_4_H_6_N_3_OS^+^·H_2_PO_4_^−^, (I), was obtained as a result of hydrolysis of [(1,3-thia­zol-2-yl­amino)­carbon­yl]­phospho­ramidic acid, (II), in water. X-ray analysis has shown that the N—P bond in (II) breaks, leading to the formation of the substituted carbamide (I). This compound exists as an inter­nal salt. The unit cell consists of a urea cation and an anion of H_2_PO_4_^−^. Protonation of the N atom of the heterocyclic ring was confirmed by the location of the H atom in a difference Fourier map. The mol­ecules of substituted urea are connected by O⋯O hydrogen bonds into unlimited planes. In turn, those planes are connected to each other *via* N—H⋯O hydrogen bonds with mol­ecules of phospho­ric acid, forming a three-dimensional polymer.

## Related literature

For background to the chemistry of phospho­rus–organic compounds, see: Ly & Woollins (1998 ▶). For details of the synthesis and properties of the [(1,3-thia­zol-2-yl­amino)­carbon­yl]phospho­ramidic acid, see: Kirsanov & Levchenko (1957 ▶); Smaliy *et al.*(2003 ▶). For structural analogues of phospho­rylated carbacyl­amides and their properties, see: Amirkhanov *et al.* (1997 ▶). For a structural investigation of phospho­rtriamidic compounds, see: Ovchynnikov *et al.* (1997 ▶). For the synthesis of the amino­thia­zol-containing phosphor­triamides, see: Shatrava *et al.* (2009 ▶). For a description of the attractive inter­action in thia­zole compounds, see: Burling & Goldstein (1992 ▶); Angyan *et al.* (1987 ▶).
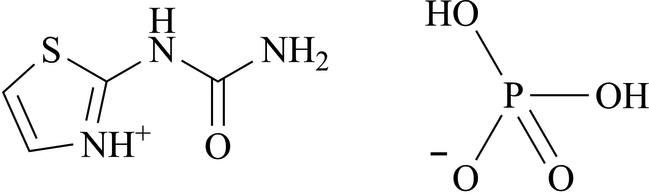


## Experimental

### 

#### Crystal data

C_4_H_6_N_3_OS^+^·H_2_PO_4_^−^
*M* *_r_* = 241.16
Monoclinic, 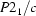

*a* = 11.9038 (11) Å
*b* = 9.7936 (10) Å
*c* = 8.1914 (12) Å
β = 97.231 (9)°
*V* = 947.37 (19) Å^3^
*Z* = 4
Mo *K*α radiation
μ = 0.51 mm^−1^
*T* = 293 K
0.30 × 0.20 × 0.20 mm

#### Data collection

Siemens SMART CCD area-detector diffractometer
Absorption correction: empirical (using intensity measurements) (*SADABS*; Bruker, 1999 ▶) *T*_min_ = 0.861, *T*_max_ = 0.904
2644 measured reflections
2239 independent reflections
1893 reflections with *I* > 2σ(*I*)
*R*_int_ = 0.014

#### Refinement

*R*[*F* ^2^ > 2σ(*F* ^2^)] = 0.038
*wR*(*F* ^2^) = 0.109
*S* = 1.05
2239 reflections
150 parameters
H atoms treated by a mixture of independent and constrained refinement
Δρ_max_ = 0.58 e Å^−3^
Δρ_min_ = −0.43 e Å^−3^

### 

Data collection: *SMART-NT* (Bruker, 1999 ▶); cell refinement: *SAINT-NT* (Bruker, 1999 ▶); data reduction: *SAINT-NT*; program(s) used to solve structure: *SHELXS97* (Sheldrick, 2008 ▶); program(s) used to refine structure: *SHELXL97* (Sheldrick, 2008 ▶); molecular graphics: *XP* within *SHELXTL* (Sheldrick, 2008 ▶); software used to prepare material for publication: *SHELXTL*.

## Supplementary Material

Crystal structure: contains datablock(s) I, global. DOI: 10.1107/S1600536811021337/dn2693sup1.cif

Structure factors: contains datablock(s) I. DOI: 10.1107/S1600536811021337/dn2693Isup2.hkl

Supplementary material file. DOI: 10.1107/S1600536811021337/dn2693Isup3.cml

Additional supplementary materials:  crystallographic information; 3D view; checkCIF report

## Figures and Tables

**Table 1:** Hydrogen-bond geometry (Å, °)

*D*—H⋯*A*	*D*—H	H⋯*A*	*D*⋯*A*	*D*—H⋯*A*
N2—H2⋯O2	0.86	1.93	2.697 (2)	148
N3—H3⋯O3	0.80 (3)	1.91 (3)	2.710 (2)	174 (3)
N1—H1*A*⋯O1^i^	0.87 (3)	2.09 (3)	2.898 (2)	155 (2)
N1—H1*B*⋯O5^ii^	0.82 (3)	2.20 (3)	3.007 (2)	170 (3)
O5—H5⋯O2^iii^	0.81 (4)	1.77 (4)	2.546 (2)	162 (4)
O4—H4⋯O3^iv^	0.80 (4)	1.82 (4)	2.613 (2)	170 (4)

Symmetry codes: (i) 
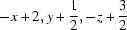
; (ii) 
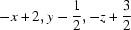
; (iii) 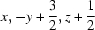; (iv) 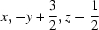.

**Fig. 1.**
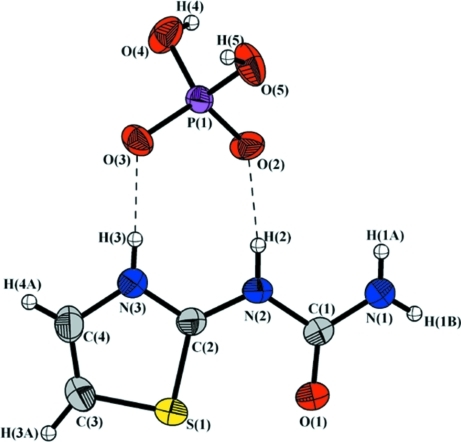
View of N-1,3-thiazol-2-yl-urea phosphate with the atom labeling scheme. Displacement ellipsoids are drawn at the 30% probability level. H atoms are represented as small spheres of arbitrary radii. Hydrogen bonds are shown as dashed lines.

**Fig. 2.**
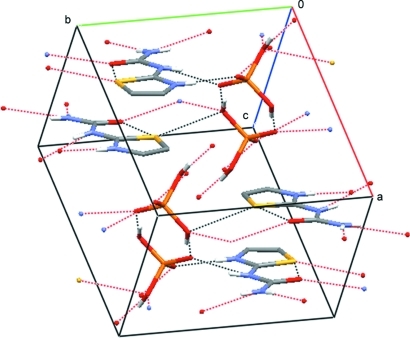
three-dimensional-view of the N-1,3-thiazol-2-yl-urea phosphate.

**Fig. 3.**
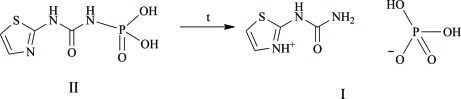
The formation of the title compound.

## supplementary crystallographic information

### Comment

The compound *N*-1,3-thiazol-2-yl-urea phosphate (I) can be synthesized by hydrolyzation of the [(1,3-thiazol-2-ylamino)carbonyl]phosphoramidic acid (II) in the water solution by heating (Scheme 1). The crystal structure investigation shows that the break up of N—P bond in [(1,3-thiazol-2-ylamino)carbonyl]phosphoramidic leads to the forming of substituted carbamide (Fig.1).

The proton of the phosphoric acid locates at the nitrogen atom of the heterocyclic ring from difference-Fourier map. The molecules of substituted urea connected by hydrogen bonds O(4)H(4)O(3) and O(5)H(5)O(2) into unlimited planes (Table 1). In turn those planes are connected to each other forming three-dimensional polymer *via* hydrogen bonds with molecules of phosphoric acid: N(3)H(3)O(3), N(2)H(2)O(2) and N(1)H(1 A)O(1), N(1)H(1B)O(5) (Table 1, Fig.2). The interaction of nonbonded S and O atoms can be described as attractive (Angyan, *et al.*, 1987). In the molecule the S O nonbonded distances are significantly shorter (2.653 Å) than the sum of the corresponding van der Waals radii (3.25 Å).

### Experimental

The synthesis of [(1,3-thiazol-2-ylamino)carbonyl]phosphoramidic acid (II) was carried out according to the method described by Kirsanov (Kirsanov & Levchenko, 1957). The compound *N*-1,3-thiazol-2-yl-urea phosphate (I) was obtained due to hydrolyzation of (II) in the water solution (Smaliy *et al.*, 2003). The crystals (I) suitable for X-ray analysis were obtained by heating of [(1,3-thiazol-2-ylamino)carbonyl]phosphoramidic acid in water and evaporating the solvent at room temperature for about 2 days.

### Refinement

H2,H3A and H4A  atoms were included in the refinement in the riding motion approximation but with refined isotropic thermal parameter. Other hydrogen atoms were refine isotropically.

### Crystal data

**Table:**

C_4_H_6_N_3_OS^+^·H_2_PO_4_^−^	*F*(000) = 496
*M**_r_* = 241.16	*D*_x_ = 1.691 Mg m^−^^3^
Monoclinic, *P*2_1_/*c*	Mo *K*α radiation, λ = 0.71073 Å
Hall symbol: -P 2ybc	Cell parameters from 2646 reflections
*a* = 11.9038 (11) Å	θ = 1.7–28.0°
*b* = 9.7936 (10) Å	µ = 0.51 mm^−^^1^
*c* = 8.1914 (12) Å	*T* = 293 K
β = 97.231 (9)°	Block, colourless
*V* = 947.37 (19) Å^3^	0.30 × 0.20 × 0.20 mm
*Z* = 4	

### Data collection

**Table:**

Siemens SMART CCD area-detector diffractometer	2239 independent reflections
Radiation source: fine-focus sealed tube	1893 reflections with *I* > 2σ(*I*)
graphite	*R*_int_ = 0.014
ω scans	θ_max_ = 28.0°, θ_min_ = 1.7°
Absorption correction: empirical (using intensity measurements) (*SADABS*; Bruker, 1999)	*h* = −14→15
*T*_min_ = 0.861, *T*_max_ = 0.904	*k* = −12→10
2644 measured reflections	*l* = −10→10

### Refinement

**Table:**

Refinement on *F*^2^	Primary atom site location: structure-invariant direct methods
Least-squares matrix: full	Secondary atom site location: difference Fourier map
*R*[*F*^2^ > 2σ(*F*^2^)] = 0.038	Hydrogen site location: inferred from neighbouring sites
*wR*(*F*^2^) = 0.109	H atoms treated by a mixture of independent and constrained refinement
*S* = 1.05	*w* = 1/[σ^2^(*F*_o_^2^) + (0.0666*P*)^2^ + 0.4134*P*] where *P* = (*F*_o_^2^ + 2*F*_c_^2^)/3
2239 reflections	(Δ/σ)_max_ = 0.001
150 parameters	Δρ_max_ = 0.58 e Å^−^^3^
0 restraints	Δρ_min_ = −0.43 e Å^−^^3^

### Special details

**Table:**

Geometry. All esds (except the esd in the dihedral angle between two l.s. planes) are estimated using the full covariance matrix. The cell esds are taken into account individually in the estimation of esds in distances, angles and torsion angles; correlations between esds in cell parameters are only used when they are defined by crystal symmetry. An approximate (isotropic) treatment of cell esds is used for estimating esds involving l.s. planes.
Refinement. Refinement of *F*^2^ against ALL reflections. The weighted *R*-factor *wR* and goodness of fit *S* are based on *F*^2^, conventional *R*-factors *R* are based on *F*, with *F* set to zero for negative *F*^2^. The threshold expression of *F*^2^ > σ(*F*^2^) is used only for calculating *R*-factors(gt) *etc*. and is not relevant to the choice of reflections for refinement. *R*-factors based on *F*^2^ are statistically about twice as large as those based on *F*, and *R*- factors based on ALL data will be even larger.

### Fractional atomic coordinates and isotropic or equivalent isotropic displacement parameters (Å^2^)

**Table:**

	*x*	*y*	*z*	*U*_iso_*/*U*_eq_	
S1	0.76064 (4)	0.11536 (5)	0.89426 (7)	0.03929 (16)	
O1	0.93971 (13)	0.18350 (15)	0.7497 (2)	0.0487 (4)	
N1	1.00646 (16)	0.3959 (2)	0.7104 (3)	0.0450 (4)	
C1	0.93508 (16)	0.30721 (18)	0.7630 (2)	0.0343 (4)	
P1	0.71692 (4)	0.70375 (4)	0.89068 (5)	0.02801 (15)	
N2	0.84692 (14)	0.36706 (16)	0.8367 (2)	0.0341 (3)	
H2	0.8438	0.4545	0.8448	0.061 (8)*	
C2	0.76722 (15)	0.29001 (18)	0.8948 (2)	0.0302 (4)	
O2	0.77229 (12)	0.62295 (13)	0.76649 (16)	0.0368 (3)	
N3	0.68142 (13)	0.34568 (18)	0.9603 (2)	0.0341 (3)	
C3	0.63845 (18)	0.1230 (2)	0.9884 (3)	0.0456 (5)	
H3A	0.5988	0.0467	1.0173	0.076 (9)*	
O3	0.66290 (12)	0.61762 (13)	1.01208 (16)	0.0357 (3)	
C4	0.60814 (17)	0.2511 (2)	1.0144 (3)	0.0414 (4)	
H4A	0.5446	0.2745	1.0636	0.062 (8)*	
O4	0.62286 (13)	0.79913 (17)	0.80225 (19)	0.0460 (4)	
O5	0.80967 (14)	0.79873 (16)	0.98184 (19)	0.0457 (4)	
H1A	1.001 (2)	0.483 (3)	0.731 (3)	0.042 (6)*	
H1B	1.054 (3)	0.359 (3)	0.660 (3)	0.053 (8)*	
H3	0.676 (2)	0.427 (3)	0.969 (3)	0.041 (6)*	
H4	0.633 (3)	0.815 (4)	0.710 (5)	0.080 (11)*	
H5	0.800 (3)	0.806 (4)	1.077 (4)	0.077 (11)*	

### Atomic displacement parameters (Å^2^)

**Table:**

	*U*^11^	*U*^22^	*U*^33^	*U*^12^	*U*^13^	*U*^23^
S1	0.0400 (3)	0.0256 (2)	0.0531 (3)	0.00031 (17)	0.0089 (2)	0.00314 (18)
O1	0.0451 (8)	0.0277 (7)	0.0769 (11)	0.0041 (6)	0.0221 (8)	−0.0017 (7)
N1	0.0420 (9)	0.0316 (9)	0.0659 (12)	0.0008 (7)	0.0243 (9)	−0.0012 (8)
C1	0.0320 (9)	0.0295 (9)	0.0424 (10)	0.0045 (7)	0.0085 (7)	0.0015 (7)
P1	0.0337 (3)	0.0256 (3)	0.0268 (2)	0.00013 (16)	0.01202 (17)	0.00036 (15)
N2	0.0372 (8)	0.0241 (7)	0.0433 (8)	0.0027 (6)	0.0138 (7)	0.0017 (6)
C2	0.0316 (8)	0.0272 (9)	0.0320 (8)	0.0023 (6)	0.0052 (7)	0.0016 (6)
O2	0.0511 (8)	0.0299 (7)	0.0328 (7)	0.0094 (5)	0.0179 (6)	0.0025 (5)
N3	0.0340 (8)	0.0306 (8)	0.0390 (8)	0.0012 (6)	0.0099 (6)	0.0002 (6)
C3	0.0366 (10)	0.0407 (11)	0.0605 (13)	−0.0074 (8)	0.0107 (9)	0.0076 (9)
O3	0.0473 (8)	0.0300 (7)	0.0328 (6)	−0.0065 (5)	0.0171 (6)	−0.0001 (5)
C4	0.0321 (9)	0.0472 (12)	0.0461 (11)	−0.0024 (8)	0.0102 (8)	0.0043 (9)
O4	0.0461 (8)	0.0575 (10)	0.0377 (8)	0.0196 (7)	0.0185 (6)	0.0120 (6)
O5	0.0513 (9)	0.0550 (10)	0.0337 (7)	−0.0228 (7)	0.0174 (6)	−0.0071 (6)

### Geometric parameters (Å, °)

**Table:**

S1—C2	1.7122 (18)	N2—C2	1.345 (2)
S1—C3	1.732 (2)	N2—H2	0.8600
O1—C1	1.218 (2)	C2—N3	1.329 (2)
N1—C1	1.324 (3)	N3—C4	1.383 (3)
N1—H1A	0.87 (3)	N3—H3	0.80 (3)
N1—H1B	0.82 (3)	C3—C4	1.330 (3)
C1—N2	1.403 (2)	C3—H3A	0.9300
P1—O2	1.5048 (12)	C4—H4A	0.9300
P1—O3	1.5083 (13)	O4—H4	0.80 (4)
P1—O5	1.5602 (16)	O5—H5	0.81 (4)
P1—O4	1.5642 (15)		
			
C2—S1—C3	89.80 (10)	C1—N2—H2	119.4
C1—N1—H1A	121.0 (16)	N3—C2—N2	121.65 (17)
C1—N1—H1B	113 (2)	N3—C2—S1	111.93 (14)
H1A—N1—H1B	126 (3)	N2—C2—S1	126.41 (14)
O1—C1—N1	125.79 (19)	C2—N3—C4	113.74 (18)
O1—C1—N2	119.90 (17)	C2—N3—H3	120.7 (19)
N1—C1—N2	114.29 (17)	C4—N3—H3	125.5 (18)
O2—P1—O3	114.27 (8)	C4—C3—S1	111.84 (15)
O2—P1—O5	107.05 (9)	C4—C3—H3A	124.1
O3—P1—O5	110.65 (8)	S1—C3—H3A	124.1
O2—P1—O4	110.48 (8)	C3—C4—N3	112.69 (18)
O3—P1—O4	107.43 (8)	C3—C4—H4A	123.7
O5—P1—O4	106.73 (10)	N3—C4—H4A	123.7
C2—N2—C1	121.12 (16)	P1—O4—H4	112 (3)
C2—N2—H2	119.4	P1—O5—H5	110 (3)
			
O1—C1—N2—C2	0.6 (3)	N2—C2—N3—C4	−179.63 (17)
N1—C1—N2—C2	179.39 (19)	S1—C2—N3—C4	0.8 (2)
C1—N2—C2—N3	−177.96 (17)	C2—S1—C3—C4	0.44 (19)
C1—N2—C2—S1	1.6 (3)	S1—C3—C4—N3	−0.1 (3)
C3—S1—C2—N3	−0.69 (15)	C2—N3—C4—C3	−0.4 (3)
C3—S1—C2—N2	179.74 (18)		

### Hydrogen-bond geometry (Å, °)

**Table:**

*D*—H···*A*	*D*—H	H···*A*	*D*···*A*	*D*—H···*A*
N2—H2···O2	0.86	1.93	2.697 (2)	148.
N3—H3···O3	0.80 (3)	1.91 (3)	2.710 (2)	174 (3)
N1—H1A···O1^i^	0.87 (3)	2.09 (3)	2.898 (2)	155 (2)
N1—H1B···O5^ii^	0.82 (3)	2.20 (3)	3.007 (2)	170 (3)
O5—H5···O2^iii^	0.81 (4)	1.77 (4)	2.546 (2)	162 (4)
O4—H4···O3^iv^	0.80 (4)	1.82 (4)	2.613 (2)	170 (4)

Symmetry codes: (i) −*x*+2, *y*+1/2, −*z*+3/2; (ii) −*x*+2, *y*−1/2, −*z*+3/2; (iii) *x*, −*y*+3/2, *z*+1/2; (iv) *x*, −*y*+3/2, *z*−1/2.
